# A randomized controlled behavioral intervention trial to improve medication adherence in adult stroke patients with prescription tailored Short Messaging Service (SMS)-SMS4Stroke study

**DOI:** 10.1186/s12883-015-0471-5

**Published:** 2015-10-21

**Authors:** Ayeesha Kamran Kamal, Quratulain Shaikh, Omrana Pasha, Iqbal Azam, Muhammad Islam, Adeel Ali Memon, Hasan Rehman, Masood Ahmed Akram, Muhammad Affan, Sumaira Nazir, Salman Aziz, Muhammad Jan, Anita Andani, Abdul Muqeet, Bilal Ahmed, Shariq Khoja

**Affiliations:** Stroke Services, Section of Neurology, Department of Medicine, The International Cerebrovascular Translational Clinical Research Training Program (Fogarty International Center, National Institutes of Health) and Aga Khan University, Stadium Road, 74800 Karachi, Pakistan; Fogarty Cerebrovascular Research Fellow, The International Cerebrovascular Translational Clinical Research Training Program (Fogarty International Center, National Institutes of Health) and Aga Khan University, Karachi, Pakistan; Epidemiology and Biostatistics Program, Department of Community Health Sciences, Aga Khan University, Karachi, Pakistan; Department of Community Health Sciences, Biostatistics, Aga Khan University, Karachi, Pakistan; SMS4Stroke Study, The International Cerebrovascular Translational Clinical Research Training Program (Fogarty International Center, National Institutes of Health) and Aga Khan University, Karachi, Pakistan; Stroke Service, Aga Khan University, Karachi, Pakistan; eHealth Innovation, Global, eHealth Resource Center, Aga Khan Development Network, Karachi, Pakistan; Epidemiology and Biostatistics, Department of Medicine, Aga Khan University, Karachi, Pakistan; Tech4Life Enterprises, and Technical Advisor-Evidence, Capacity & Policy mHealth Alliance, United Nations Foundation, Washington, USA

**Keywords:** Stroke, Medication adherence, SMS, Prevention, Non communicable disease, mHealth, IT technology, Lower and middle income countries, Cost effectiveness

## Abstract

**Background:**

The effectiveness of mobile technology to improve medication adherence via customized Short Messaging Service (SMS) reminders for stroke has not been tested in resource poor areas. We designed a randomized controlled trial to test the effectiveness of SMS on improving medication adherence in stroke survivors in Pakistan.

**Methods:**

This was a parallel group, assessor-blinded, randomized, controlled, superiority trial. Participants were centrally randomized in fixed block sizes. Adult participants on multiple medications with access to a cell phone and stroke at least 4 weeks from onset (Onset as defined by last seen normal) were eligible. The intervention group, in addition to usual care, received reminder SMS for 2 months that contained a) Personalized, prescription tailored daily medication reminder(s) b) Twice weekly health information SMS. The Health Belief Model and Social Cognitive theory were used to design the language and content of messages. Frontline SMS software was used for SMS delivery. Medication adherence was self-reported and measured on the validated Urdu version of Morisky Medication Adherence Questionnaire. Multiple linear regression was used to model the outcome against intervention and other covariates. Analysis was conducted by intention-to-treat principle.

**Results:**

Two hundred participants were enrolled. 38 participants were lost to follow-up. After 2 months, the mean medication score was 7.4 (95 % CI: 7.2–7.6) in the intervention group while 6.7 (95 % CI: 6.4–7.02) in the control group. The adjusted mean difference (Δ) was 0.54 (95 % CI: 0.22–0.85). The mean diastolic blood pressure in the intervention group was 2.6 mmHg (95 % CI; −5.5 to 0.15) lower compared to the usual care group.

**Conclusion:**

A short intervention of customized SMS can improve medication adherence and effect stroke risk factors like diastolic blood pressure in stroke survivors with complex medication regimens living in resource poor areas.

**Trial registration:**

Clinicaltrials.gov NCT01986023 last accessed at https://clinicaltrials.gov/ct2/show/NCT01986023

**Electronic supplementary material:**

The online version of this article (doi:10.1186/s12883-015-0471-5) contains supplementary material, which is available to authorized users.

## Background

Stroke is the second major cause of death and third largest contributor to disability globally [1, 2]. Two thirds of this burden is borne by low and middle income countries where they are more likely to be fatal or disabling [3]. International comparison of stroke cost studies show that on average, stroke care accounted for 3 % of total health care expenditures [4]. In Pakistan, community surveys suggest a lifetime stroke symptom prevalence of approximately 19 % [5], with an estimated annual stroke incidence of 250 per 100,000 population [6, 7].

Optimal adherence to medications may reduce the risk of poor outcomes by 26 % [8]. However, a recent 50 year (1948–1998) meta-analysis reported global adherence rates around 75 % [9]. Local studies report adherence rates to cardiac medicines ranging between 27–77 % [10] and a 68 % compliance in stroke patients in the first 2 years after the event [11].

Interventions designed to overcome non-adherence include drug diaries, pill counters, automated reminders, patient counseling and improving social support [12–15]. Each of these interventions, involves substantial cost, time and effort with a variable response dependent on health and prescription literacy and self-motivation [16]. These are not feasible in settings like Pakistan due to poor health literacy and awareness and severe resource limitations [17, 18]. Short text message (SMS) is an inexpensive, ubiquitous and culturally acceptable tool with potential for behavioral change. Mobile phone users in Pakistan were recorded at greater than 137 million by the Pakistan Telecommunication Authority and total cellular density is reportedly 77 % [19]. We hypothesized that our short intense SMS intervention would be able to demonstrate its potential if we used the Health Belief Model with Behavioural Change Theory to design it and reach large numbers frequently due to economic feasibility [20–22].

We sought to determine the effectiveness of customized SMS reminders plus Health information SMS in addition to usual care in adult stroke patients compared to usual care only in improving medication adherence at a hospital stroke service in Pakistan. In addition we explored the biologic effects if any, on blood pressure for those who received SMS and the scalability characteristics of the innovation based on Rogers Diffusion of Innovation Theory derived questionnaire that measures intervention qualities such as Simplicity, Compatibility, Observability and Relative advantage [23].

## Methods

SMS for Stroke is a parallel-group, assessor-blinded, randomized controlled single center superiority trial conducted to assess the intervention of SMS reminders on adherence [24]. The participants are randomized into two parallel groups in a 1:1 ratio via block technique with one group receiving the standard of care as per institutional guidelines while the parallel group receiving SMS reminders for each dose of medicine in addition to the standard of care. Following is a brief outline of the methodology used in the study. For further details and access to questionnaires and tools, please refer to our paper on trial methods [23].

### Study setting

The SMS for Stroke Study was conducted at the Clinical Trials Unit (CTU); Aga Khan University which is a JCIA (Joint Commission International Accreditation) accredited hospital in Karachi, Pakistan. Stroke service is delivered at the center through a 24 h neurovascular team on floor and ambulatory care clinics.

### Participants

Participants were recruited from the Neurology and Stroke Clinics at this tertiary care center. The average daily volume of the center is 100+ Visits and annual volumes are greater than 1500+ patients to the single stroke clinic alone.

### Eligibility criteria

#### Inclusion criteria

- Age greater than 18 years old
- History of stroke(s) confirmed by neuroimaging at the time of the episode
- >1 month since last episode of stroke
- Use of at least two drugs such as (but not limited to) anti platelets, statins, anti-hypertensives to control risk factors of stroke.
- Modified Rankin Score of 3 or less (so that they are able to operate mobile phones)
- Possession of a personal cell phone that the patient has access to at all times. In the case of patients who do not own or are unable to use mobile phones, they must have a caregiver available at all times who possesses a cell phone.
- Ability to receive, comprehend and reply to an SMS in English, Nastaleeq Urdu (local Urdu script) or Roman Urdu. In the case of patients who themselves are unable to receive, comprehend or reply to an SMS, they must have caregivers available at all times who could perform the above mentioned tasks.

#### Exclusion criteria

- Biological impairment in reading or responding to SMS in the caregiver such as (but not limited to) loss of vision, visual field cuts, aphasia in case the patient himself/herself is supposed to receive SMS
- Diagnosed organ dysfunction or malignancy such as hepatic, renal or malignancy
- Plans to travel outside the country inside the two months following enrollment

### Assignment of interventions

Centrally Randomised computer generated sequence was used by the CTU and allocation concealed in opaque white envelopes. Participants were assigned to groups in a parallel fashion in a 1:1 ratio. Block randomization technique was used with block size of 10 (not disclosed to field and research team that was directly interacting with participants). This is to ensure similarity between the two groups at all times permitting interim analysis during the study.

### Study procedures

Participants were invited after assessment of eligibility and those who consented were interviewed in the CTU regarding demographic information, medical and prescription details. The baseline Morisky adherence score for each patient was also recorded at this time followed by the randomisation to either the treatment group A or intervention group B. After allocation, the research supervisor explained the details of the intervention to the participants in group A and demonstrated by sending one test SMS on his/her cell phone in the preferred language for SMS. Since the participants were required to respond via SMS, all participants were compensated for the cost of sending the response by providing them with prepaid credit in advance. In case of allocation to usual care group, the participants were informed about their date of follow up after 2 months. The staff who randomized and those who assessed and those who delivered the intervention were separate.

### Control group

In the control group, patients receive the usual standard of care provided at the center for stroke patients. This primarily consists of regular follow up visits (as advised by their neurologist) with their stroke neurologist. In general, these are at 1, 3, 5,9,12 months after a stroke. Each patient is provided with a telephone number that can be used to reach the stroke team in case of an emergency and each patient is also reminded of their clinic appointments 1–2 days prior via SMS and/or phone.

### Intervention group

**Fig. 1:**
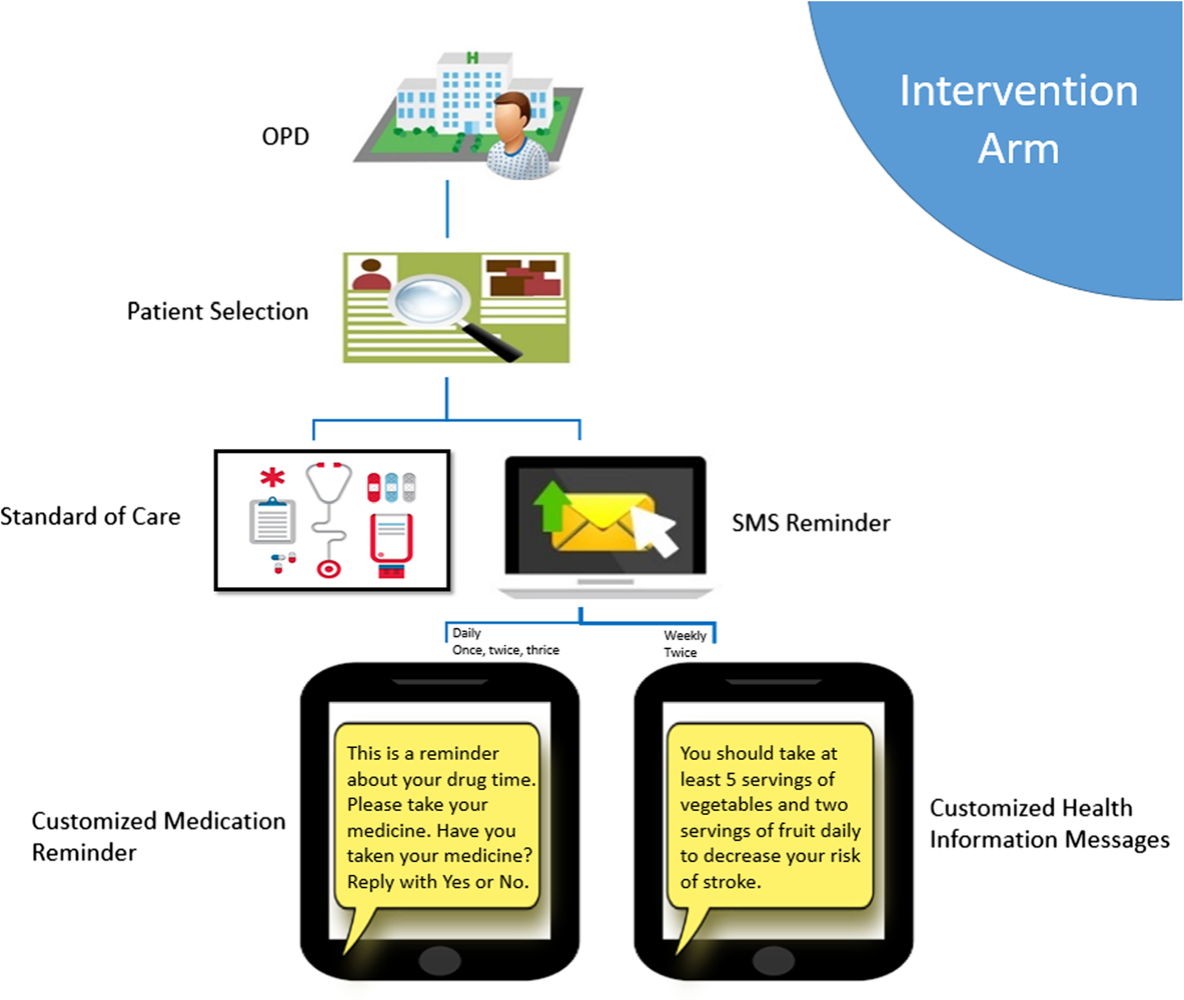
Information flow diagram

In addition to the usual care, intervention group received automated SMS reminders customized to their individual prescription. The participants were required to respond to the SMS stating if they have taken their medicines. Moreover twice weekly health information SMS were also sent to the intervention group. Health information SMS were customized according to medical and drug profile of every patient by the research team. The messages were designed in a weekly schedule at preset days of the week for total 8 weeks e.g., Wednesday and Saturday week 1 for patient X. The timings were decided according to the prescription so that health messages do not collide with the reminder messages for that day. Usually 5 pm was found feasible for most participants. These messages did not ask for a reply. These health information SMS were codified by Michie’s Taxonomy of Behavioural Change for repeatability [25] (Fig. 1).

### Follow up and outcome ascertainment

The subjects were required to follow up after 2 months in the CTU. In order to improve the follow up rate participants in both groups were sent SMS and reminded about their due follow up 1–2 days earlier. If the participant was not able to report exactly after 2 months, a period of ± 7 days was allowed for adjusting the follow up. Those participants who did not appear for follow up were contacted on phone up to 3 times and also approached by other means like sending transport to aid them or contacting them when they come for other clinic visit, lab work, physiotherapy etc. All participants were compensated for their travelling cost. Outcome assessment was performed by trained study physicians who were masked to the group that the participant was assigned. In addition, separate assessors evaluated participant response to SMS intervention. Enrollment began on 5th December 2013 and the last patient was recruited on 30th April 2014. The last followup was conducted on 28th June 2014.

### Outcomes

#### Primary outcome measure

The primary outcome of interest was a change in medication adherence after 2 months of receiving the SMS. Medication adherence was measured at recruitment and after 2 months in both groups on the Morisky Medication Adherence Scale (MMAS). The scale has been used in a similar setting previously and it has been translated and validated in Urdu [26]. The instrument consists of 8 questions and the response to first 7 questions is scored as either 0 or 1. The eighth question has a weighted response from 0.25 to 1. The tool has a sensitivity of 46 % and specificity of 60 % for the Urdu version which is the lingua franca of the population, and this version has been validated [26].

#### Secondary outcome measures

##### Effect on biologic variables: blood pressure

We explored the effect, if any, on blood pressure, even if though the intervention was short term. Blood pressure was measured via Mindray Datascope Equator in the CTU at registration visit and after interview to assess for variability due to stress with the participant sitting and relaxed.

##### SMS intervention assessment

We also measured patient satisfaction and acceptability of using an innovation such as the SMS to improve clinical outcome. This was done through tools which identified the beneficial and untoward attributes of using this technology. One of the tools was a self-reported questionnaire originally designed keeping in mind Roger’s factors from his theory of Diffusion of Innovations and measured patient satisfaction as a percentage [27, 28]. Another questionnaire was designed based on previous literature which measured patient satisfaction and was also reported as proportions [29].

### Ethics and human subjects protection

All patients taking part in the trial were required to provide written informed consent at the time of recruitment. Consent forms were available in English and Urdu. Special care was taken to send the health promotional texts twice weekly at times that do not cause discomfort to the patient such as late at night. Our messages did not contain identifying information and the program sending the messages was secure at a single site with limited access. All staff received requisite GCP training and credentials. The participants were compensated for their travel and phone expenses. A hotline was created for patient queries and concerns. The study was approved by the Ethical Research Committee, Aga Khan University, Pakistan with approval number 2763-Med-ERC-13.

## Plan of analysis and sample size

Based on literature, we estimated the mean MMAS score to be 6 [30] in the control group and 7 in the intervention group, giving a mean difference of 1 (SD = 2). Using these values, a sample of at least 172 subjects was required to achieve a power of 90 % and significance level of 5 % when testing a two tailed hypothesis of inequality of means. This translated into 16 % effect size. Keeping a 15 % attrition rate the sample size was inflated so at least 100 subjects were needed in each group. Any improvement in the MMAS would translate into clinical improvement in the long run through effective secondary prevention.

Pilot Testing was done on 10 % of the sample size i.e., 20 participants and the intervention was also tested for smooth application and any systematic errors. This sample was excluded from the final analysis. These 20 participants were in addition to the 200 participants that were included in the final study.

Data was entered on Microsoft Access database through double entry. Analysis was performed using the intention to treat principle at two stages: interim analysis after 25 % of the sample had been reached (Additional file 1) and final analysis after data had been collected from all study participants. Descriptive statistics were reported as Mean (SD) or Median (IQR) for continuous variables like age, years of schooling, years since diagnosis, MMAS score etc. Proportions were reported for categorical variables like gender, marital status, area of residence, employment status, proportion of patients with depression etc. Multiple linear regression was performed to estimate the adjusted mean difference in MMAS between the two groups. Robust regression was applied to the final model. Sensitivity analysis were done by duration of intervention, SMS receiver (patient/caregiver) and primary stroke physician (refer to Additional file 1). The acceptability and patient satisfaction of the intervention were reported as proportions. Stata version 12 was used for analysis.

An interim analysis was performed to ensure that the IT based technology was not causing any unexpected outcomes that were not foreseen, in addition to ensure that the program was being delivered with fidelity.

## Results

**Fig. 2:**
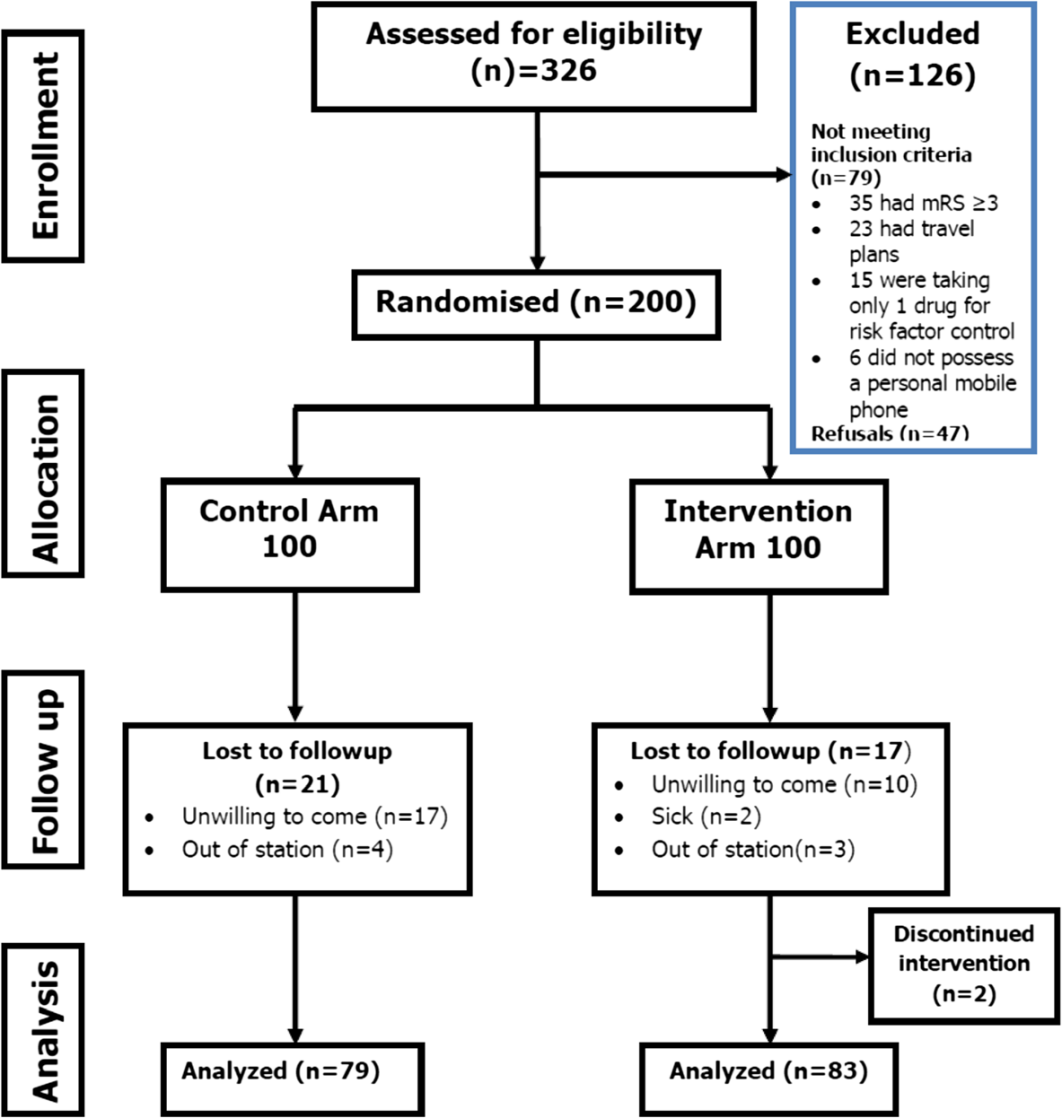
Study flow chart. mRS-modified Rankin Scale. Out of Station = Not in the city and unable to report for follow up during the period that outcome assessment was supposed to be performed. Discontinued Intervention = Withdrew from the study and were not sent SMS, they did not want to have SMS sent to them

Three hundred twenty six patients were approached for enrollment where 126 were excluded due to ineligibility or lack of consent (Fig. 2). One hundred participants were randomised to each group. After 2 months, 21 were lost to followup in control group while 19 in intervention group.

### Baseline characteristics (Table 1)

A total of 200 participants were analyzed in the study (100 in each group). Of these, 135 (67.5 %) were male while 65 (32.5 %) were female. There were fewer males (64) in the control group as compared to the intervention group (71). The mean age in the intervention group was lower (56 years. S.D 1.5 years) compared to (57.6 years, S.D 1.3 years) in the usual care group. These differences were not statistically significant.

**Table 1:** Baseline characteristics of the study participants

		Intervention group	Usual care group
		* n* = 100	*n* = 100
		n (%)	n (%)
1	Age (years)^a^	56.07 (1.5)	57.62 (1.3)
2	Male	64 (64)	71 (71)
3	Educated	90 (90)	88 (88)
4	Years of formal education^a^	12.7 (0.4)	12.36 (0.4)
5	Urban residence	86 (86)	88 (88)
6	Number of pills prescribed daily^b^	7 (4.5–9.5)	8 (6–10)
7	Distance from stroke physician^b^ (km)	9.9 (7.2–16.7)	11.6 (7.8–18.6)
8	Monthly cost of drugs^b^ (PKR)	12,000 (7500–18,000)	12,000 (7500–19,750)
9	Side effects	13 (13)	12 (12)
10	Use of alternate medicines	12 (12)	12 (12)
11	Missed physician appointments in last year	13 (13)	14 (14)
12	Dosing frequency once daily	5 (5)	4 (4)
	Twice daily	60 (60)	66 (66)
	Thrice daily	35 (35)	30 (30)
13	Use of pill boxes	15 (15)	16 (16)
14	Use of alarms as medication reminders	3 (3)	2 (2)
15	Baseline Morisky adherence score^b^	7 (5.7–8)	7 (5.7–8)
16	Blessed dementia score^b^	4.5 (2–7.2)	4 (2.5–7)
17	Social support scale^b^	12 (7–18)	12 (6–18)
18	Ischemic stroke	83 (83)	84 (84)
19	Time since stroke^b^	2 (1–5)	2 (1–4)

There were no statistically significant baseline differences in the study participants

^a^Mean (SD)

^b^Median (IQR)

### Mean medication adherence

The baseline median Morisky medication adherence score was similar in the two groups (6.6). After 2 months of follow-up, the MMAS increased in both groups. While the increase was minor in the control group (+0.1), there was a much larger increase in the intervention group (+0.8). This difference was found to be statistically significant (Table 2). On univariate analysis the mean medication adherence score was 0.65 (0.0–1.0) points higher in the intervention group compared to the usual care group (Table 3). It was observed that high number of pills prescribed daily, high monthly cost of drugs, higher level of social support, missing physician appointments in the previous year, ischemic stroke and presence of depression were all inversely related with the level of medication adherence. Since cost of drugs was skewed it was log transformed to obtain linearity with the outcome. The baseline Morisky adherence score, being unemployed or retired, being educated and higher dosing frequency were positively related to the level of medication adherence. Multivariable analysis showed that mean difference in adherence score between the intervention group and the usual care group was 0.54 (95 % CI; 0.22–0.85) (*p* = <0.01) adjusted for all other variables. (Table 2) The model explains 30 % of variability in the outcome (r^2^ = 0.3).

**Table 2:** Mean Morisky medication adherence score at baseline and 2-month follow-up in Intervention vs. usual care group (multivariable analysis)

	Intervention group^a^		Usual care group^a^		Adjusted difference^b^ (95 % CI)
	Baseline	2 months	Baseline	2 months	
Morisky medication adherence score	6.6 (0.17)	7.4 (0.93)	6.6 (0.16)	6.7 (1.32)	0.54 (0.22–0.85)*

*p < 0.01

^a^Mean (SD)

^b^adjusted for baseline adherence score, number of pills prescribed daily, dosing frequency, age, gender, employment status, education, use of alarms, missing physician appointments in the previous year and block design

**Table 3:** Factors associated with adherence to medications (univariate analysis)

		β coefficient	95 % CI	p value
1	Intervention	0.65	0.3–1.0	0.00
2	Number of pills prescribed daily	−0.0475	−0.09 to–0.03	0.04
3	Social support scale	−0.01266	−0.03 to–0.01	0.21
4	Baseline Morisky adherence scale	0.2644	0.15 to 0.38	0.00
5	Educated	0.363	−0.25 to 0.97	0.24
6	Missed appointments in last year	−0.518	−1.05 to 0.013	0.05
7	Dosing frequency of medicines once daily	Ref		
	Twice daily	0.868	−0.03 to 1.76	0.06
	Thrice daily	0.466	−0.46 to 1.39	0.32
8	Use of alarms	0.955	−0.40 to 2.31	0.16
9	Ischemic stroke	−0.329	−0.821 to 0.161	0.19

### Secondary outcomes

#### Biologic effects – blood pressure

We limited our exploratory outcomes to the change in systolic and diastolic blood pressure of participants. This was due to the fact that major biologic changes were not reasonably expected with such limited exposure to intervention. Although no major effect was observed on systolic blood pressure after the intervention (change of 1 mm of Hg *p* = 0.678), the diastolic blood pressure did show a significant change over a 2 month period. The mean diastolic blood pressure in the intervention group was 2.6 mmHg (95 % CI; −5.5 to 0.15: *p* = 0.06) lower compared to the control group after the intervention (Table 4).

**Table 4:** Effect on mean diastolic blood pressure

	Mean DBP	Mean DBP	Mean difference	95 %(CI)*
	Pre-intervention	Post-intervention		
	mmHg	mmHg		
Intervention group	80	77.9	−2.6	−5.5 to 0.15
Usual care group	80.6	80.5	−0.1	

*DBP* diastolic blood pressure

*p = 0.06

### Acceptability of intervention

#### 1. Patient satisfaction with intervention

The overall mean score for this tool was 12.5 out of 13 which is equivalent to a mean percentage of 96.07 % (Table 5).

**Table 5:** Patient satisfaction with intervention

	Mean (SD)/total	Mean %
Patient satisfaction with intervention	12.5 (1.5)/13	96.07 %

#### 2. Diffusion characteristics of mHealth (mobile health) intervention

The overall mean score for this outcome was 7.6 out of 8 which translates into an overall mean percentage score of 95.6 % (Table 6). The four attributes of Roger’s Diffusion theory were scored separately. The score for Simplicity was 1.91/2, Compatibility was 1.91/2, Observability was 1.9/2 and Relative advantage was 1.95/2.

**Table 6:** Acceptability of mHealth intervention

	Mean (SD)/total	Mean %
Acceptability of mHealth intervention	7.6 (1.1)/8	95.6 %
Components		
Simplicity	1.91 (0.35)/2	95.5 %
Compatibility	1.91 (0.35)/2	95.5 %
Observability	1.90 (0.40)/2	95 %
Relative advantage	1.95 (0.21)/2	97.5 %

## Discussion

This study is an early report of an SMS based intervention for improving medication adherence in stroke survivors based in a low resource setting. The results show a significant increase in medication adherence behavior which is encouraging and highlights the possibility of improving secondary stroke prevention through a simple intervention. Additionally, a small but significant difference in diastolic blood pressure was observed in those who received SMS, who were presumably more compliant in the intervention group. Users of the SMS for stroke service reported a high satisfaction and acceptability and the intervention itself showed good characteristics as an innovation that may disseminate favorably.

We observed that the dosing frequency had a positively linear relationship with mean medication adherence in the presence of intervention. This shows that the intervention was effective in achieving high adherence for participants with most difficult dosing regimens like thrice daily frequency. This was possible because the intervention was tailored to individual patient prescription and reminders were sent according to dosing schedule. On the other hand it was seen that mean adherence was inversely related to total pill count prescribed per day. This contrasts with previous findings from a study in Pakistani hypertensive population [10], where increasing number of pills increased the adherence scores. Higher pill count may lead to patient fatigue and poorer adherence. Patients with stroke are have relative cognitive impairment leading to poorer adherence to a complex prescriptions, however in spite of this setting the intervention was effective and users became more compliant.

mHealth (mobile Health) is a rapidly developing field whose potential of leverage to improve medically important outcomes is great but is limited by a lack of well-designed randomized controlled trials that measure robust outcomes [31, 32]. Most SMS studies are focused on communicable diseases with a recent shift towards non-communicable diseases. SMS based interventions have shown modest effect [33–41]. We feel that in addition to robust RCT design the actual SMS wordings of our intervention were designed on theories of behavior change and may explain some effect as compared to simple knowledge transfer messages [42]. Most IT interventions are not informed by theory or frameworks that would explain the mechanisms of why a message would work or not, and be replicable by other teams. We used the Health Belief Model, as opposed to simple knowledge transfer, which predicts influences on human behavior have 6 key determinants: *Perceived susceptibility*, *Perceived seriousness*, *Perceived benefits of taking action*, *Barriers to taking action, Cues to Action and Self-efficacy* [43, 44]. Thus participants were enabled to change their behavior via messages that touched on these themes.

Adherence has two components namely: i) Intentional non-adherence and ii) non-intentional non-adherence. It is important to distinguish the contribution of both the types in order to devise successful interventions [45]. We targeted both aspects by providing knowledge and belief change messages and the other by cueing, nudging and reminder behavior to take medications.

The major strength of this study is its RCT design, with allocation concealment, blinded outcome ascertainment, and use of validated tools and effort to reduce attrition. We used an open access software for designing the intervention. Our intervention is clear, designed and taxonomy coded and replicable. Furthermore, sensitivity analyses also reinforce the independent effect of intervention.

The main limitation of this trial is the use of self-reported outcome measure the validated Morisky Medication Adherence Scale (MMAS), which was chosen due to the complex stroke medication regimen and population characteristics. There have been comparative studies where self-reported adherence measures, like questionnaires, are found to be acceptable compared to more objective methods of measuring drug adherence like electronic pill boxes, biomarkers [46–48]. We considered the use of electronic pill boxes and biomarkers for outcome assessment. However, stroke patients have diverse prescriptions which vary in the type of drug classes, number and frequency of dosage, no single biomarker would be applicable to all the study participants. Similarly, electronic pill boxes record the number of times a box is opened. Since stroke patients are on multiple drugs at any dosing time, it would be erroneous to believe that they have consumed all the pills for that dose when they open the box. It was logistically difficult to request disabled participants to physically visit for repeated pill counts. So we relied on self-reported scale as a measure of adherence. Additionally, to counter check our measure, we documented day to day adherence with return SMS from the participant. There are no ideal measures for reporting adherence, the MMAS itself is a reliable measure of self-reported adherence as it corresponds well to pharmacy refill rates [49, 50]. We are exploring phone based adherence measures to improve our adherence measurement outcomes in future studies, such as unannounced pill counts and capsule photographs [51, 52]. Another limitation is that the duration of this study did not allow measurement of definite biologic outcomes like stroke recurrence after the intervention. It may be argued that the patient population for this study had minimal disability (MRS < 3), but it is this high risk group which should be saved from recurrent disability by stroke recurrence. Moreover, our eligibility data show that 77 % of the stroke patients coming to our clinic were eligible for this intervention and only 11 % were excluded due to disability (Fig. 2). An inherent limitation of the study is the performance bias of an educational intervention; participants were not blinded to the reception of SMS and were well instructed and probably motivated to medication adherence than the control group. This motivation may be partially responsible for some of the adherence behavior. Although our population may have poor literacy and health literacy skills overall, we used a short text message service to improve adherence due to the fact that Roman Urdu (easily legible) and Bolo SMS (Verbal SMS) options were used in the study to send messages to participants and this is what helped with acceptability and reach. Pakistanis have exchanged 301.7 billion SMS with 317 million users during 2014, covering 92 % of the land area. In this study, there was an expected limitation for those who would not possess a cell phone, based on the eligibility criteria and review, 6 of the 326 potential participants (1.8 %) were excluded on this basis and we found that at least based on mobile infrastructure related basis, we were able to reach out to 98 % of the population at our center. Despite our efforts to keep the loss of follow up minimal, we did experience a 20 % loss to follow up due to hesitation and limitation of disabled persons to travel for follow up. In future interventions, we are planning Skype™ assisted teleconference follow ups to measure functional status and other outcomes of interest in disabled persons.

Some operational difficulties with the intervention were faced when the SMS service was blocked in the country for security reasons. There were 3 separate occasions when this happened, each lasting 24 h. We anticipated such events and informed our participants about the possibility of not being able to send reminder messages that day and that they should manage their medications themselves to avoid anxiety. After set up it was very easy to operate the system.

The effect on diastolic blood pressure needs to be strengthened by improving study power and duration of exposure. We believe this tool has the potential to bring such changes if used over a significant duration.

The potential impact of using SMS to cue chronic disease desirable behavior is immense. SMS is incredibly popular and acceptable, with Pakistani mobile phone users exchanging a staggering 315.7 billion text messages during July 2012 to June 2013 or 865 million SMS messages a day and prefer this mode of communication [39]. There are 135 million registered phone users in Pakistan whose data are biometrically verified, in addition there is a national electronic database where all users are linked and registered and potentially able to receive SMS [19]. The cost of SMS is cheap including bundles for business and social marketing, and the software used to deploy mass messages is open access and freely available. Stroke happens a decade earlier in LMIC countries like Pakistan and the population at risk is using cell phones. Additionally, since the stroke survivor and primary caregiver reside in communities, the primary caregiver often has access to a mobile phone and it is possible to make the intervention effective in a relatively older population. Additionally, although the population has literacy challenges a text based reminder system works even in those who have had minimal schooling due to the widespread understanding of Roman Urdu (which we also used to send our messages).

Although it is not known how SMS would affect terminal outcomes like recurrent strokes, death or disability, it is known that the effect size itself is modest. In spite of these acknowledged limitations, in population dense regions, in absolute numbers, millions of lives could be reached and positively influenced regardless of geopolitical strife, chaos, socioeconomic differences and access inequities.

## Conclusions

In conclusion, we feel that the SMS intervention seems feasible for clinical use in stroke survivors for improving adherence. Further studies are needed to report on meaningful biologic outcomes like recurrent stroke, death and disability. Cost effectiveness, scalability characteristics beyond what we have reported, are also areas in need of further research exploration, as larger scale policy informing analysis.

## Additional file

Additional file 1:
**Interim Analysis.** (DOC 37 kb)

## Abbreviations

SMS: Short text message
AKUH: Aga Khan university hospital
JCIA: Joint commission international accreditation
CTU: Clinical trials unit
MMAS: Morisky medication adherence scale
mHealth: Mobile health
