# Supplementary material for: A randomized controlled behavioral intervention trial to improve medication adherence in adult stroke patients with prescription tailored Short Messaging Service (SMS)-SMS4Stroke study
Source: BMC Neurol. 2015 Oct 21;15:212. doi: 10.1186/s12883-015-0471-5 (PMC4618367; doi:10.1186/s12883-015-0471-5)
Supplement: Additional file 1: — Interim Analysis. (DOC 37 kb) [file 12883_2015_471_MOESM1_ESM.doc]

**Interim Analysis**

# Interim analysis was performed when follow-ups were completed on 25% of the study sample (50 participants). There were 25 participants in each arm with 23 males and 2 females in the control group while 17 males and 8 females in the intervention group. Attrition rate was found to be 28%.(Table 2)

# The baseline mean Morisky score in the control arm was 5.6 (0.36) and 6.2(0.38) in intervention arm while after the intervention it increased to 6.1 (0.32) and 7.2 (0.23) respectively. Hence mean adherence score was 0.79 (β) (p=0.04) higher in the intervention arm compared to usual care arm when adjusted for other variables in the model. (r^2^=0.37).

# Sensitivity Analysis:

# Sensitivity analysis was done by primary stroke physician, duration of intervention and who received the SMS (patient or caregiver). There was no change in results by these factors (Tables 5-7). There was less than 2% missing information on only two variables, i.e. source of coverage for health care and number of elderly persons above 65 years of age in the participant’s home.

Table 2: Mean difference in adherence score between the 2 groups (Interim Data)

|  | **Intervention Arm^†^ n=50** | | **Usual care Arm^†^ n=50** | | **Adjusted difference^*^ (95% CI)** |
| --- | --- | --- | --- | --- | --- |
|  | Baseline | 2 Months | Baseline | 2 Months |  |
| **Morisky Medication Adherence Score** | 6.2 (0.38) | 7.2(0.23) | 5.6(0.36) | 6.1(.32) | 0.79(0.03-1.5)**^*#^** |

**†**Mean (SD)

*****adjusted for baseline Morisky adherence score, age and gender

#p<0.05

# SENSITIVITY ANALYSIS

Table 5: Model adjusted for duration of intervention

|  | **Adjusted difference*** | **95% CI** | **p value** |
| --- | --- | --- | --- |
| **Intervention** | 0.47 | 0.15-0.80 | <0.01 |

*adjusted for baseline adherence score, number of pills prescribed daily, dosing frequency, age, gender, employment status, education, use of alarms, missing physician appointments in the previous year and block design + duration of intervention

Table 6: Model adjusted for SMS receiver (patient/caregiver)

|  | **Adjusted difference*** | **95% CI** | **p value** |
| --- | --- | --- | --- |
| **Intervention** | 0.48 | 0.16-0.81 | <0.01 |

*adjusted for baseline adherence score, number of pills prescribed daily, dosing frequency, age, gender, employment status, education, use of alarms, missing physician appointments in the previous year and block design + SMS receiver

Table 7: Model adjusted by Primary Stroke physician

|  | **Adjusted difference*** | **95% CI** | **p value** |
| --- | --- | --- | --- |
| **Intervention** | 0.46 | 0.13-0.79 | <0.01 |

*adjusted for baseline adherence score, number of pills prescribed daily, dosing frequency, age, gender, employment status, education, use of alarms, missing physician appointments in the previous year and block design + primary stroke physician
